# Supplementary material for: New Insights on the Effect of TNF Alpha Blockade by Gene Silencing in Noise-Induced Hearing Loss
Source: Int J Mol Sci. 2020 Apr 13;21(8):2692. doi: 10.3390/ijms21082692 (PMC7215896; doi:10.3390/ijms21082692)
Supplement: Supplementary file 1 [file ijms-21-02692-s001.pdf]

Table S1. Gene expression (qRT-PCR) obtained without (no noise) and with noise exposure.

Targets	Ct (no noise)	Ct (noise)	2^dCt	Fold change 1/2^dCt noise
18S	6.525	6.988	0.928	-1.078
ACTB	17.155	17.522	0.992	-1.008
AGFG1	21.712	22.332	0.832	-1.201
APAF1	25.149	25.760	0.838	-1.194
ARHGDIB	23.359	23.450	1.201	1.201
B2M	18.605	18.633	1.255	1.255
BAG4	23.271	24.010	0.766	-1.305
BCL2	24.700	25.776	0.607	-1.648
BID	26.128	26.192	1.224	1.224
CAD	23.527	23.969	0.942	-1.062
CASP2	24.180	24.981	0.734	-1.362
CASP3	24.115	24.153	1.246	1.246
CASP8	24.704	26.489	0.371	1.300
CD27	32.197	33.106	0.681	-1.468
CD40	30.165	30.194	1.254	1.254
CD40LG	29.553	30.644	0.600	-1.750
CD70	33.807	34.971	0.571	-1.752
CFLAR	21.887	22.551	0.807	-1.239
CHUK	24.883	25.254	0.989	-1.011
CYCS	20.401	21.208	0.731	-1.368
DFFA	23.415	24.204	0.740	-1.351
DUSP1	24.702	26.252	0.437	-2.289
FADD	24.774	25.650	0.697	-1.435
FAF1	22.418	22.973	0.871	-1.149
FAZ	27.567	27.866	1.040	1.040
FASLG	26.855	27.522	0.806	-1.241
FBF1	22.868	23.461	0.848	-1.179
GADPH	17.781	17.985	1.110	1.110
GUSB	24.234	24.602	0.991	-1.009
HMBS	24.674	24.982	1.033	1.033
HPRT1	22.168	22.801	0.825	-1.212
IKBKAP	22.616	23.225	0.839	-1.192
IKBKB	23.839	24.340	0.904	-1.106
IKBKG	23.670	24.168	0.906	-1.104
JUN	21.785	22.036	1.075	1.075
LMNA	22.199	22.262	1.224	1.224
LMNB1	25.351	25.254	1.368	1.368
LTA	32.969	33.097	1.171	1.171
LTB	28.363	29.368	0.637	-1.569

LTBR	23.666	24.145	0.918	-1.080
MADD	23.531	24.278	0.762	-1.312
MAP2K4	21.759	22.420	0.809	-1.236
MAP3K1	24.640	22.718	4.847	4.847
MAP3K14	24.504	27.188	0.199	-5.024
MAP3K7	26.627	24.806	4.520	4.520
MAPK8	24.414	25.235	0.724	-1.381
NFKB1	23.560	24.308	0.762	-1.313
NFKBIA	22.539	24.273	0.385	-2.601
NGFR	23.608	23.923	1.028	1.028
NGFRAP1	21.290	21.959	0.805	-1.243
PAK1	22.001	22.426	0.953	-1.050
PAK2	20.776	21.253	0.919	1.08
PARP1	21.017	21.625	0.839	-1.192
PGK1	19.144	19.743	0.845	-1.184
PGLYRP1	26.285	27.244	0.658	-1.520
PSMG2	23.454	24.075	0.832	-1.202
RB1	22.788	23.126	1.012	1.012
RELT	25.751	26.742	0.644	-1.554
RIPK1	24.988	25.568	0.856	-1.169
RPLP2	20.165	20.497	1.016	1.016
RPLPO	19.493	19.968	0.920	-1.087
SPTAN1	18.525	19.370	0.712	-1.404
TAB1	26.282	27.035	0.759	-1.318
TANK	23.844	24.657	0.728	-1.373
TBP	24.553	25.227	0.802	-1.247
TFRC	20.680	21.857	0.566	-1.768
TNF	29.158	28.467	2.065	2.065
TNFRSF10B	31.968	32.249	1.053	1.053
TNFRSF11B	34.145	34.632	0.913	-1.096
TNFRSF12A	28.987	29.198	1.105	1.105
TNFRSF14	29.528	30.334	0.732	-1.367
TNFRSF17	35.508	38.194	0.199	-5.020
TNFRSF1A	19.724	20.212	0.912	-1.096
TNFRSF1B	26.932	27.416	0.915	-1.093
TNFRSF21	26.959	27.339	0.983	-1.017
TNFRSF25	20.123	20.285	-0.875	-1.090
TNFRSF4	25.036	25.256	1.098	1.098
TNFRSF8	22.957	23.069	1.184	1.184
TNFRSF9	26.180	26.740	0.868	-1.153
TNFSF10	30.724	31.379	0.812	-1.900
TNFSF11	32.655	32.965	1.032	1.032

TNFSF12	25.577	26.858	0.526	-1.900
TNFSF13	23.024	23.373	0.723	-1.310
TNFSF14	23.024	23.774	0.761	-1.315
TNFSF15	33.482	33.206	1.549	1.549
TNFSF18	27.040	27.864	0.723	-1.700
TNFSF4	32.962	34.085	0.587	-1.230
TNFSF9	29.064	29.503	0.944	-2.365
TRADD	25.219	26.192	0.652	-1.535
TRAF2	24.160	24.875	0.779	-1.283
TRAF3	23.048	23.770	0.775	-1.290
TRAF4	25.734	26.370	0.823	-1.215
TRAF6	23.353	23.963	0.838	-1.193
TRAF7	23.797	24.260	0.928	-1.07
UBC	19.264	20.029	0.753	-1.329
YWHAZ	19.248	19.457	1.107	1.107

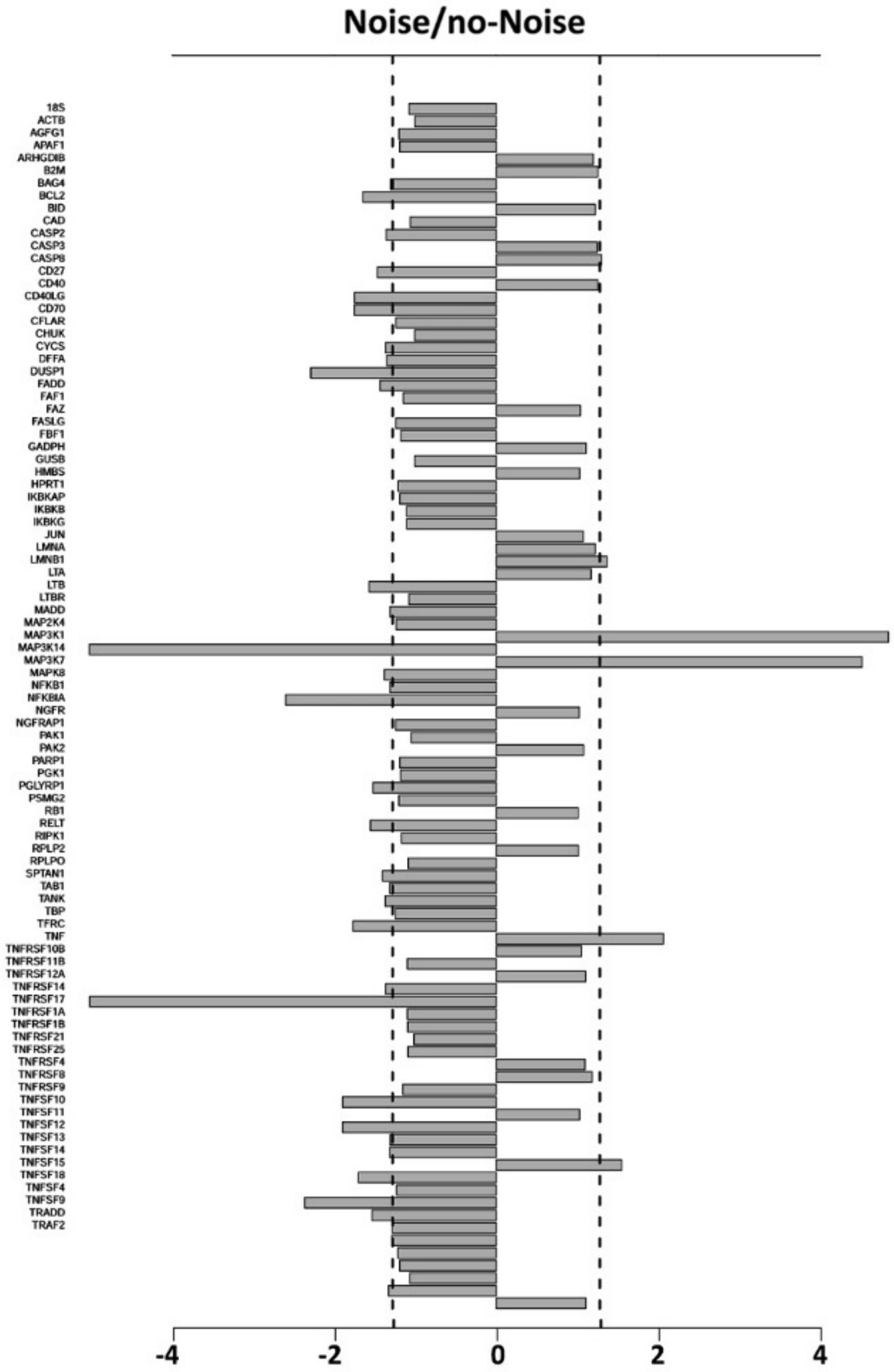

Figure S1. Gene expression (qRT-PCR) obtained without (no noise) and with noise exposure.

Table S2. Gene expression (qRT-PCR) obtained after noise exposure without (scramble) and with gene silencing (*Tnf-α* siRNA).

Targets	Ct (scrambled)	Ct (*Tnf-α* siRNA)	2^dCt	Fold change 1/2^dCt siRNA
18S	7,371	6,489	1,241	
ACTB	17,513	17,251	0,808	-1,238
AGFG1	22,729	22,211	0,965	-1,030
APAF1	25,963	25,523	0,914	-1,094
ARHGDIB	23,526	23,681	0,605	-1,653
B2M	18,178	18,895	0,410	-2,440
BAG4	24,578	23,689	1,248	1,248
BCL2	26,189	26,41	0,578	-1,730
BID	25,945	26,027	0,636	-1,571
CAD	24,346	24,199	0,746	-1,341
CASP2	25,201	24,084	1,461	1,461
CASP3	24,631	24,404	0,788	-1,268
CASP8	25,93	26,075	0,609	-1,641
CD27	31,115	32,61	0,239	-4,184
CD40	29,338	30,155	0,382	-2,615
CD40LG	31,716	34,081	0,131	-7,647
CD70	31,639	31,211	0,906	-1,103
CFLAR	22,316	22,245	0,708	-1,413
CHUK	25,216	25,026	0,768	-1,301
CYCS	21,456	21,127	0,846	-1,182
DFFA	24,321	24,377	0,648	-1,543
DUSP1	25,42	25,526	0,626	-1,598
FADD	26,362	25,07	1,650	1,650
FAF1	23,261	22,77	0,947	-1.05
FAZ	26,953	27,807	0,373	-2,683
FASLG	27,873	27,514	0,864	-1,157
FBF1	24,895	23,632	1,617	1,617
GADPH	18,016	17,445	1,001	1,001
GUSB	25,256	24,662	1,017	1,017
HMBS	25,153	24,604	0,986	-1.02
HPRT1	23,409	23,005	0,891	-1,122
IKBKAP	23,916	23,135	1,158	1,158
IKBKB	24,437	23,836	1,022	1,022
IKBKG	24,804	24,084	1,110	1,110
JUN	22,762	22,089	1,074	1,074
LMNA	22,776	21,976	1,173	1,173
LMNB1	25,754	25,303	0,921	-1.08
LTA	32,098	32,873	0,394	-2,540

LTB	27,122	27,523	0,510	-1,960
LTBR	24,677	23,852	1,193	1,193
MADD	24,687	23,853	1,201	1,201
MAP2K4	22,917	22,005	1,268	1,268
MAP3K1	25,172	24,277	1,253	1,253
MAP3K14	27,289	26,48	1,180	1,180
MAP3K7	22,754	22,169	1,011	1,011
MAPK8	25,288	24,819	0,932	-1,070
NFKB1	24,021	23,906	0,730	-1,371
NFKBIA	22,362	23,123	0,398	-2,516
NGFR	24,522	23,984	0,978	-1.02
NGFRAP1	22,293	21,667	0.962	-1.04
PAK1	23,295	19,614	8,640	8,640
PAK2	21,513	20,982	0,973	-1.02
PARP1	22,412	21,41	1,349	1,349
PGK1	20,266	19,769	0,951	-1.04
PGLYRP1	25,304	26,798	0,239	-4,181
PSMG2	24,604	24,023	1,008	1,008
RB1	23,323	22,955	0,869	-1,150
RELT	26,543	25,914	1,042	1,042
RIPK1	25,438	25,254	0,765	-1,307
RPLP2	20,403	19,991	0,896	-1,116
RPLPO	20,968	20,595	0,872	-1,146
SPTAN1	20,181	19,132	1,394	1,394
TAB1	27,615	26,594	1,367	1,367
TANK	24,682	24,223	0,926	-1.033
TBP	25,793	25,17	1,037	1,037
TFRC	21,839	21,238	1,022	1,022
TNF	26,185	27,562	0,259	-3,856
TNFRSF10B	27,461	27,458	0,675	-1,481
TNFRSF11B	20,421	20,335	0,715	-1,399
TNFRSF12A	24,87	24,948	0,638	-1,567
TNFRSF14	27,783	29,488	0,207	-4,840
TNFRSF17	35,558	33,924	2,091	2,091
TNFRSF1A	22,937	22,973	0,657	-1,522
TNFRSF1B	23,774	25,578	0,193	-5,183
TNFRSF21	20,564	19,778	1,162	1,162
TNFRSF25	28,277	27,142	1,479	1,479
TNFRSF4	30,249	31,189	0,351	-2,848
TNFRSF8	33,722	35,178	0,246	-4,073
TNFRSF9	28,944	28,947	0,672	-1,488
TNFSF10	26,734	25,861	1,234	1,234

TNFSF11	29,424	29,936	0,472	-2,117
TNFSF12	23,443	24,399	0,347	-2,880
TNFSF13	23,163	23,958	0,388	-2,576
TNFSF14	31,249	32,506	0,282	-3,548
TNFSF15	28,229	27,944	0,821	-1,218
TNFSF18	32,227	32,518	0,551	-1,816
TNFSF4	32,042	31,72	0,842	-1,187
TNFSF9	32,415	33,087	0,423	1,032
TRADD	26,972	25,966	1,353	1,353
TRAF2	25,638	23,871	2,293	2,293
TRAF3	23,928	23,315	1,030	1,030
TRAF4	26,419	25,781	1,048	1,048
TRAF6	24,146	23,621	0,969	-1.03
TRAF7	25,112	24,351	1,142	1,142
UBC	20,252	19,757	0,949	1.05
YWHAZ	19,606	19,163	0,916	-1,092
PCL2	25,188	26,41	0,289	-3,463

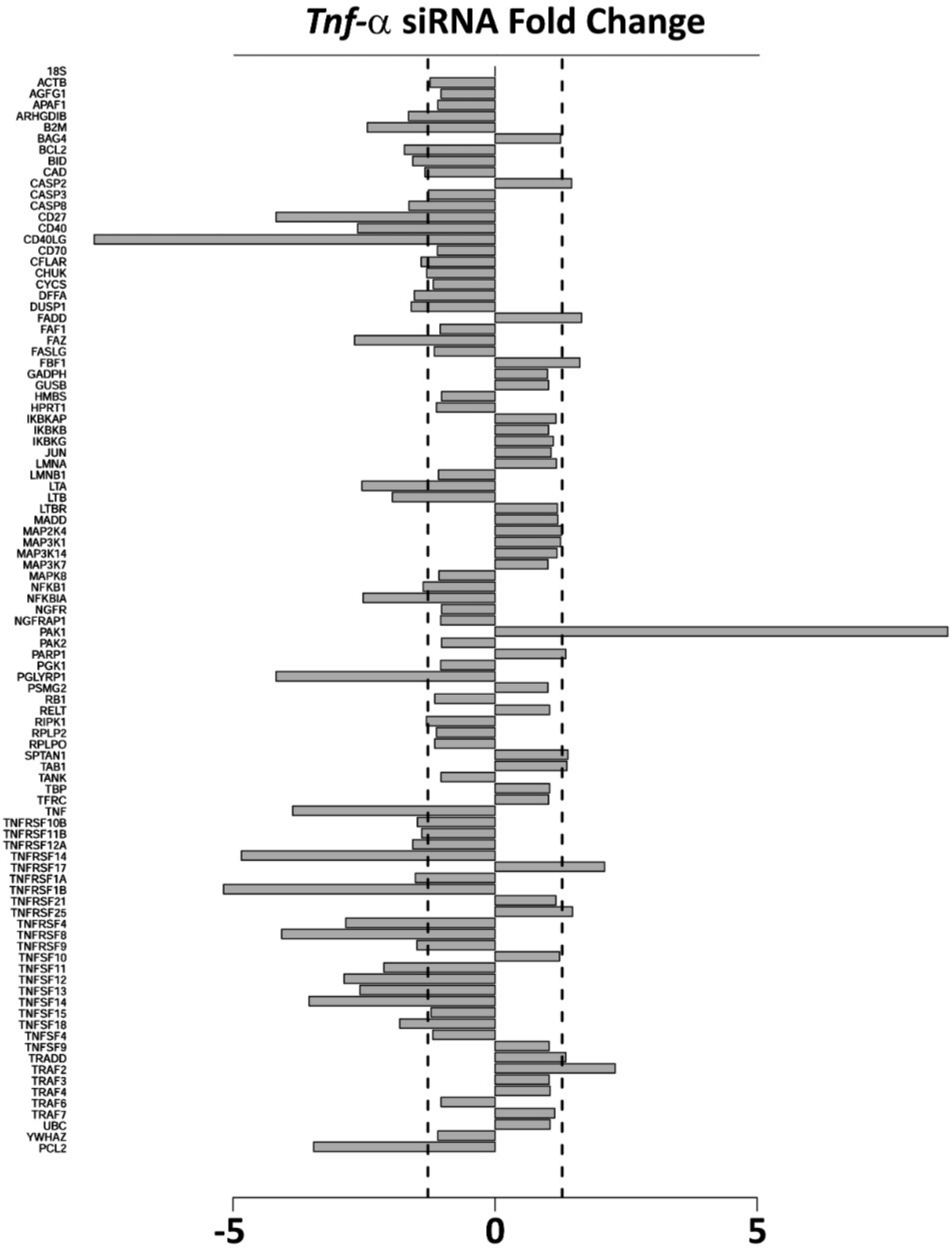

Figure S2. Gene expression (qRT-PCR) obtained after noise exposure without (scramble) and with gene silencing (*Tnf-α* siRNA).
